# 2-(4-Methoxy­phen­yl)-4,5-dihydro-1*H*-imidazole

**DOI:** 10.1107/S1600536809009106

**Published:** 2009-03-19

**Authors:** Reza Kia, Hoong-Kun Fun, Hadi Kargar

**Affiliations:** a X-ray Crystallography Unit, School of Physics, Universiti Sains Malaysia, 11800 USM, Penang, Malaysia; b Department of Chemistry, School of Science, Payame Noor University (PNU), Ardakan, Yazd, Iran

## Abstract

In the title mol­ecule, C_10_H_12_N_2_O, the dihedral angle between the benzene and imidazole rings is 14.86 (16)°. The approximately planar arrangement of the mol­ecule results in a distance of 2.54 Å between an *ortho*-H atom of the benzene ring and the double-bonded N atom of the imidazole ring. In the crystal structure, symmetry-related mol­ecules are linked by inter­molecular N—H⋯N hydrogen bonds into one-dimensional chains extending along the *a* axis.

## Related literature

For hydrogen-bond motifs, see: Bernstein *et al.* (1995 ▶). For related structures and syntheses, see: Stibrany *et al.* (2004 ▶); Kia *et al.* (2008 ▶, 2009*a* ▶,*b* ▶). For applications, see, for example: Blancafort (1978 ▶); Chan (1993 ▶); Vizi (1986 ▶); Li *et al.* (1996 ▶); Ueno *et al.* (1995 ▶); Corey & Grogan (1999 ▶). For details on the stability of the temperature controller used for data collection, see: Cosier & Glazer (1986 ▶). For bond-length data, see: Allen *et al.* (1987 ▶).
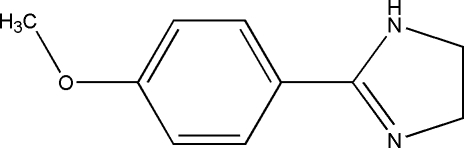


## Experimental

### 

#### Crystal data

C_10_H_12_N_2_O
*M* *_r_* = 176.22
Orthorhombic, 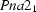

*a* = 10.0574 (5) Å
*b* = 13.2532 (7) Å
*c* = 6.8321 (3) Å
*V* = 910.67 (8) Å^3^
*Z* = 4
Mo *K*α radiation
μ = 0.09 mm^−1^
*T* = 100 K
0.23 × 0.09 × 0.06 mm

#### Data collection

Bruker SMART APEXII CCD area-detector diffractometer
Absorption correction: multi-scan (**SADABS**; Bruker, 2005 ▶) *T*_min_ = 0.981, *T*_max_ = 0.995
8578 measured reflections
1133 independent reflections
873 reflections with *I* > 2σ(*I*)
*R*_int_ = 0.049

#### Refinement

*R*[*F* ^2^ > 2σ(*F* ^2^)] = 0.046
*wR*(*F* ^2^) = 0.096
*S* = 1.08
1133 reflections
123 parameters
1 restraint
H atoms treated by a mixture of independent and constrained refinement
Δρ_max_ = 0.22 e Å^−3^
Δρ_min_ = −0.22 e Å^−3^

### 

Data collection: *APEX2* (Bruker, 2005 ▶); cell refinement: *APEX2*; data reduction: *SAINT* (Bruker, 2005 ▶); program(s) used to solve structure: *SHELXTL* (Sheldrick, 2008 ▶); program(s) used to refine structure: *SHELXTL*; molecular graphics: *SHELXTL*; software used to prepare material for publication: *SHELXTL* and *PLATON* (Spek, 2009 ▶).

## Supplementary Material

Crystal structure: contains datablocks global, I. DOI: 10.1107/S1600536809009106/lh2787sup1.cif

Structure factors: contains datablocks I. DOI: 10.1107/S1600536809009106/lh2787Isup2.hkl

Additional supplementary materials:  crystallographic information; 3D view; checkCIF report

## Figures and Tables

**Table 1:** Hydrogen-bond geometry (Å, °)

*D*—H⋯*A*	*D*—H	H⋯*A*	*D*⋯*A*	*D*—H⋯*A*
N1—H1N1⋯N2^i^	0.93 (3)	1.95 (3)	2.869 (3)	168 (3)

Symmetry code: (i) 
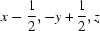
.

**Fig. 1.**
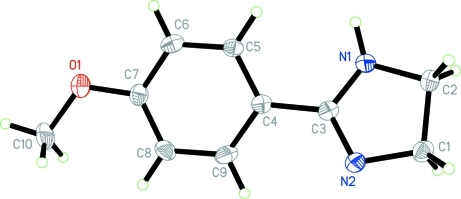
The molecular structure of the title compound with atom labels and 50% probability ellipsoids for non-H atoms.

**Fig. 2.**
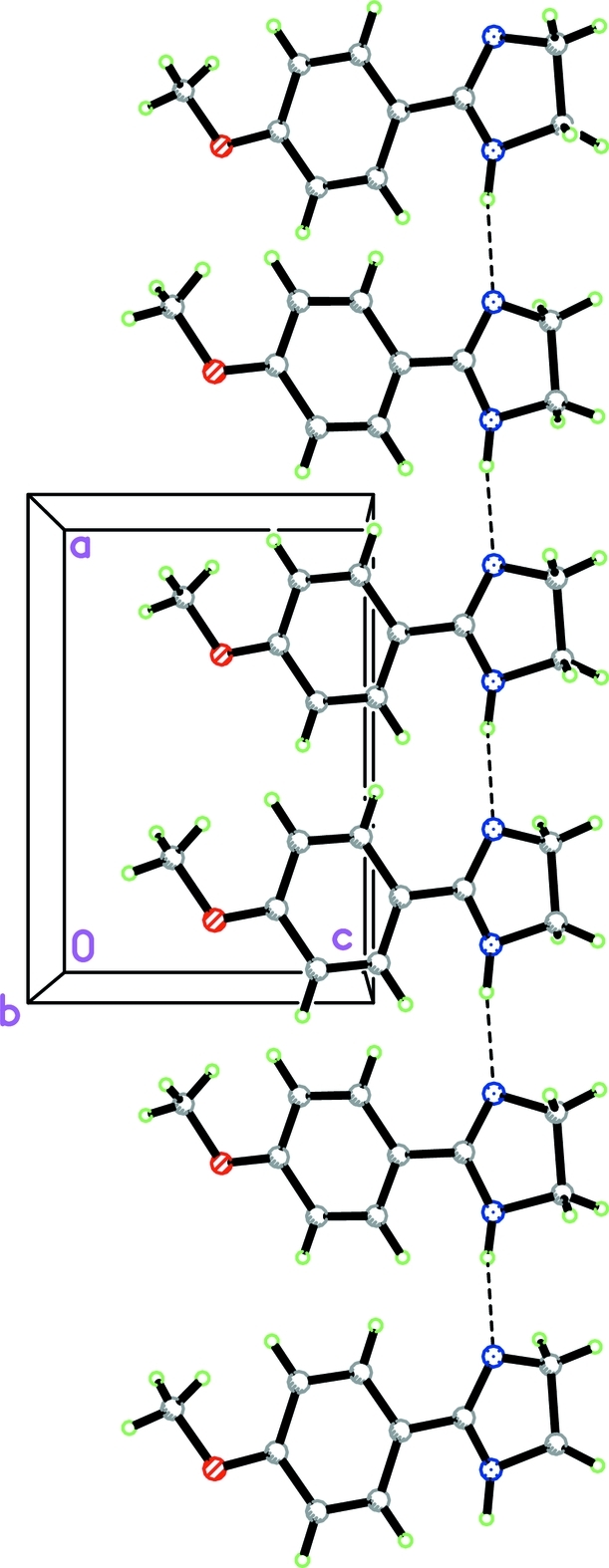
Part of the crystal structure of the title compound, viewed along the b-axis showing a 1-D extended chain along the a-axis formed by intermolecular N—H···N hydrogen bonds (dashed lines).

## supplementary crystallographic information

### Comment

Imidazoline derivatives are of great importance because they exhibit significant biological and pharmacological activities such as antihypertensive (Blancafort 1978), antihyperglycemic (Chan 1993), antidepressive (Vizi 1986), antihypercholesterolemic (Li *et al.*, 1996) and anti-inflammatory (Ueno *et al.*, 1995) properties. These compounds are also used as catalysts and synthetic intermediates in some organic reactions (Corey & Grogan 1999). With regards to these important applications of imidazolines, herein we report the crystal structure of the title compound, (I).

In the title compound (I, Fig. 1), bond lengths (Allen *et al.* 1987) and angles are with the normal ranges and are comparable with the related structures (Stibrany *et al.* 2004; Kia *et al.*, 2008, 2009*a*,b). The molecule is approximately planar with a maximum deviation from the mean plane of the molecule for atom N1 being 0.279 (2) Å. The six- and five-membered rings are twisted from each other, forming the dihedral angle of 14.86 (16)°. Atom H5A of the benzene ring is in close proximity to atom N2 atom of the imidazoline ring with a distance of 2.54 Å [N2···H5A]. In the crystal structure, neighbouring molecules are linked together by intermolecular N—H···N hydrogen bonds into 1-D extended chains along the *a* axis (Table 1, Fig. 2).

### Experimental

The synthetic method was based on the previous work (Stibrany *et al.* 2004), except that 10 mmol of 4-methoxy-cyanobenzene and 40 mmol of ethylenediamine was used. Single crystals suitable for *X*-ray diffraction were obtained by evaporation of an methanol solution at room temperature.

### Refinement

The N-bound hydrogen atom was located from the difference Fourier map are refined freely, see Table. 1. The rest of the hydrogen atoms were positioned geometrically with a riding approximation model with C—H = 0.95–0.99 Å and *U*_iso_(H) = 1.2 & 1.5 *U*_eq_(C). A rotating group model was applied for the methyl group. In the absence of significant anomalous dispersion effects, 943 Friedel pairs were merged before the final refinement.

### Crystal data

**Table:**

C_10_H_12_N_2_O	*F*(000) = 376
*M**_r_* = 176.22	*D*_x_ = 1.285 Mg m^−^^3^
Orthorhombic, *P**n**a*2_1_	Mo *K*α radiation, λ = 0.71073 Å
Hall symbol: P 2c -2n	Cell parameters from 2309 reflections
*a* = 10.0574 (5) Å	θ = 2.5–30.0°
*b* = 13.2532 (7) Å	µ = 0.09 mm^−^^1^
*c* = 6.8321 (3) Å	*T* = 100 K
*V* = 910.67 (8) Å^3^	Block, colourless
*Z* = 4	0.23 × 0.09 × 0.06 mm

### Data collection

**Table:**

Bruker SMART APEXII CCD area-detector diffractometer	1133 independent reflections
Radiation source: fine-focus sealed tube	873 reflections with *I* > 2σ(*I*)
graphite	*R*_int_ = 0.049
φ and ω scans	θ_max_ = 27.5°, θ_min_ = 2.5°
Absorption correction: multi-scan (*SADABS*; Bruker, 2005)	*h* = −13→12
*T*_min_ = 0.981, *T*_max_ = 0.995	*k* = −12→17
8578 measured reflections	*l* = −8→8

### Refinement

**Table:**

Refinement on *F*^2^	Primary atom site location: structure-invariant direct methods
Least-squares matrix: full	Secondary atom site location: difference Fourier map
*R*[*F*^2^ > 2σ(*F*^2^)] = 0.046	Hydrogen site location: inferred from neighbouring sites
*wR*(*F*^2^) = 0.096	H atoms treated by a mixture of independent and constrained refinement
*S* = 1.08	*w* = 1/[σ^2^(*F*_o_^2^) + (0.0375*P*)^2^ + 0.2936*P*] where *P* = (*F*_o_^2^ + 2*F*_c_^2^)/3
1133 reflections	(Δ/σ)_max_ < 0.001
123 parameters	Δρ_max_ = 0.22 e Å^−^^3^
1 restraint	Δρ_min_ = −0.22 e Å^−^^3^

### Special details

**Table:**

Experimental. The crystal was placed in the cold stream of an Oxford Cyrosystems Cobra open-flow nitrogen cryostat (Cosier & Glazer, 1986) operating at 100.0 (1) K.
Geometry. All e.s.d.'s (except the e.s.d. in the dihedral angle between two l.s. planes) are estimated using the full covariance matrix. The cell e.s.d.'s are taken into account individually in the estimation of e.s.d.'s in distances, angles and torsion angles; correlations between e.s.d.'s in cell parameters are only used when they are defined by crystal symmetry. An approximate (isotropic) treatment of cell e.s.d.'s is used for estimating e.s.d.'s involving l.s. planes.
Refinement. Refinement of *F*^2^ against ALL reflections. The weighted *R*-factor *wR* and goodness of fit *S* are based on *F*^2^, conventional *R*-factors *R* are based on *F*, with *F* set to zero for negative *F*^2^. The threshold expression of *F*^2^ > σ(*F*^2^) is used only for calculating *R*-factors(gt) *etc*. and is not relevant to the choice of reflections for refinement. *R*-factors based on *F*^2^ are statistically about twice as large as those based on *F*, and *R*- factors based on ALL data will be even larger.

### Fractional atomic coordinates and isotropic or equivalent isotropic displacement parameters (Å^2^)

**Table:**

	*x*	*y*	*z*	*U*_iso_*/*U*_eq_	
O1	0.6844 (2)	0.00507 (17)	−0.4396 (3)	0.0302 (6)	
N1	0.6252 (2)	0.28654 (19)	0.3242 (4)	0.0270 (6)	
N2	0.8459 (2)	0.25758 (18)	0.3284 (4)	0.0219 (5)	
C1	0.8179 (3)	0.3256 (3)	0.4948 (4)	0.0234 (7)	
H1A	0.8555	0.3935	0.4702	0.028*	
H1B	0.8571	0.2985	0.6169	0.028*	
C2	0.6653 (3)	0.3309 (3)	0.5111 (4)	0.0233 (7)	
H2A	0.6321	0.2909	0.6233	0.028*	
H2B	0.6339	0.4014	0.5236	0.028*	
C3	0.7329 (3)	0.2397 (2)	0.2447 (4)	0.0176 (6)	
C4	0.7204 (3)	0.1776 (2)	0.0662 (4)	0.0178 (6)	
C5	0.5988 (3)	0.1384 (2)	0.0039 (4)	0.0184 (6)	
H5A	0.5208	0.1512	0.0784	0.022*	
C6	0.5902 (3)	0.0813 (2)	−0.1641 (4)	0.0227 (7)	
H6A	0.5067	0.0551	−0.2044	0.027*	
C7	0.7032 (3)	0.0620 (2)	−0.2748 (4)	0.0218 (7)	
C8	0.8257 (3)	0.0985 (2)	−0.2139 (4)	0.0236 (7)	
H8A	0.9036	0.0844	−0.2876	0.028*	
C9	0.8332 (3)	0.1557 (3)	−0.0444 (4)	0.0232 (7)	
H9A	0.9172	0.1805	−0.0026	0.028*	
C10	0.7990 (3)	−0.0211 (3)	−0.5514 (4)	0.0319 (8)	
H10A	0.7722	−0.0624	−0.6637	0.048*	
H10B	0.8609	−0.0594	−0.4694	0.048*	
H10C	0.8425	0.0405	−0.5981	0.048*	
H1N1	0.537 (3)	0.266 (2)	0.310 (6)	0.043 (10)*	

### Atomic displacement parameters (Å^2^)

**Table:**

	*U*^11^	*U*^22^	*U*^33^	*U*^12^	*U*^13^	*U*^23^
O1	0.0298 (12)	0.0350 (14)	0.0258 (10)	−0.0013 (10)	0.0007 (10)	−0.0112 (10)
N1	0.0167 (13)	0.0387 (17)	0.0257 (12)	0.0020 (12)	−0.0016 (12)	−0.0098 (14)
N2	0.0188 (12)	0.0275 (14)	0.0194 (10)	−0.0002 (11)	−0.0020 (11)	−0.0019 (12)
C1	0.0236 (15)	0.0274 (18)	0.0192 (13)	−0.0035 (14)	−0.0023 (14)	0.0011 (12)
C2	0.0231 (14)	0.0282 (18)	0.0187 (13)	−0.0005 (14)	−0.0016 (13)	−0.0036 (12)
C3	0.0192 (15)	0.0186 (16)	0.0150 (11)	−0.0016 (13)	−0.0017 (12)	0.0053 (12)
C4	0.0183 (15)	0.0192 (16)	0.0157 (12)	−0.0006 (13)	−0.0003 (12)	0.0044 (13)
C5	0.0160 (14)	0.0203 (17)	0.0189 (12)	0.0011 (12)	−0.0008 (11)	0.0024 (12)
C6	0.0182 (15)	0.0253 (17)	0.0247 (13)	−0.0028 (13)	−0.0042 (13)	0.0030 (14)
C7	0.0254 (17)	0.0239 (18)	0.0159 (13)	−0.0003 (14)	−0.0004 (12)	−0.0016 (13)
C8	0.0220 (16)	0.0284 (18)	0.0204 (13)	0.0007 (13)	0.0060 (13)	−0.0007 (14)
C9	0.0177 (16)	0.0304 (19)	0.0215 (14)	−0.0056 (14)	−0.0023 (12)	0.0023 (13)
C10	0.039 (2)	0.034 (2)	0.0227 (16)	0.0027 (16)	0.0053 (14)	−0.0100 (15)

### Geometric parameters (Å, °)

**Table:**

O1—C7	1.368 (3)	C4—C9	1.393 (4)
O1—C10	1.425 (4)	C4—C5	1.396 (4)
N1—C3	1.361 (4)	C5—C6	1.377 (4)
N1—C2	1.463 (4)	C5—H5A	0.9500
N1—H1N1	0.94 (3)	C6—C7	1.389 (4)
N2—C3	1.294 (3)	C6—H6A	0.9500
N2—C1	1.478 (4)	C7—C8	1.387 (4)
C1—C2	1.541 (4)	C8—C9	1.386 (4)
C1—H1A	0.9900	C8—H8A	0.9500
C1—H1B	0.9900	C9—H9A	0.9500
C2—H2A	0.9900	C10—H10A	0.9800
C2—H2B	0.9900	C10—H10B	0.9800
C3—C4	1.476 (4)	C10—H10C	0.9800
			
C7—O1—C10	117.6 (2)	C5—C4—C3	122.2 (3)
C3—N1—C2	108.2 (2)	C6—C5—C4	120.9 (3)
C3—N1—H1N1	126 (2)	C6—C5—H5A	119.6
C2—N1—H1N1	118 (3)	C4—C5—H5A	119.6
C3—N2—C1	106.6 (2)	C5—C6—C7	120.3 (3)
N2—C1—C2	105.8 (2)	C5—C6—H6A	119.9
N2—C1—H1A	110.6	C7—C6—H6A	119.9
C2—C1—H1A	110.6	O1—C7—C8	124.2 (3)
N2—C1—H1B	110.6	O1—C7—C6	115.9 (3)
C2—C1—H1B	110.6	C8—C7—C6	119.9 (2)
H1A—C1—H1B	108.7	C9—C8—C7	119.3 (3)
N1—C2—C1	101.1 (2)	C9—C8—H8A	120.3
N1—C2—H2A	111.5	C7—C8—H8A	120.3
C1—C2—H2A	111.5	C8—C9—C4	121.5 (3)
N1—C2—H2B	111.5	C8—C9—H9A	119.2
C1—C2—H2B	111.5	C4—C9—H9A	119.2
H2A—C2—H2B	109.4	O1—C10—H10A	109.5
N2—C3—N1	116.0 (2)	O1—C10—H10B	109.5
N2—C3—C4	122.8 (3)	H10A—C10—H10B	109.5
N1—C3—C4	121.1 (2)	O1—C10—H10C	109.5
C9—C4—C5	118.1 (3)	H10A—C10—H10C	109.5
C9—C4—C3	119.7 (3)	H10B—C10—H10C	109.5
			
C3—N2—C1—C2	8.4 (3)	C3—C4—C5—C6	179.5 (3)
C3—N1—C2—C1	14.4 (3)	C4—C5—C6—C7	−0.1 (4)
N2—C1—C2—N1	−13.7 (3)	C10—O1—C7—C8	2.3 (4)
C1—N2—C3—N1	1.0 (3)	C10—O1—C7—C6	−176.7 (3)
C1—N2—C3—C4	177.3 (3)	C5—C6—C7—O1	−179.7 (2)
C2—N1—C3—N2	−10.7 (3)	C5—C6—C7—C8	1.3 (4)
C2—N1—C3—C4	172.9 (3)	O1—C7—C8—C9	179.9 (3)
N2—C3—C4—C9	−15.1 (4)	C6—C7—C8—C9	−1.2 (4)
N1—C3—C4—C9	161.0 (3)	C7—C8—C9—C4	−0.2 (4)
N2—C3—C4—C5	164.1 (3)	C5—C4—C9—C8	1.4 (4)
N1—C3—C4—C5	−19.8 (4)	C3—C4—C9—C8	−179.4 (3)
C9—C4—C5—C6	−1.3 (4)		

### Hydrogen-bond geometry (Å, °)

**Table:**

*D*—H···*A*	*D*—H	H···*A*	*D*···*A*	*D*—H···*A*
N1—H1N1···N2^i^	0.93 (3)	1.95 (3)	2.869 (3)	168 (3)

Symmetry codes: (i) *x*−1/2, −*y*+1/2, *z*.
